# 

**Published:** 1887-10

**Authors:** 


### TABLE OF CONTENTS.

### Original and Selected Articles.

Remarks on Tetanus at International Medical Congress, by J. McF Gaston, M. D., Atlanta, Georgia ..... 397
Man an Object of Zoology, by S. W. Stiles, M. D., Georgia, continued from page 359 ..... 401
Quinine—When to Give it and How to Give It ..... 402
Notes on the Value of Cocaine in Minor Surgery, by A. P. Morgan Vance, M.D ..... 404
Antipyrin in Puerperal Fever, by A B. Anderson, M. D., Pawnee City, Nebraska ..... 407
Carbuncles Successfully Treated with Carbolic Acid and Salicylic Acid Injections, by C. H. Wilkinson, M. D ..... 410
Dietary in Catarrh of the Stomach ; The Treatment of Epistaxis; M. Gallippe ..... 412

### Abstracts and Gleanings.

Proper Time to ive Medicines ..... 413
Hygiene of the Hair ..... 414
Pasteur and his New Communication ..... 415
Nitro-glycerine; Heroic Dose of Turpentine in Croup ..... 416
The Modern Treatment of Urethritis ..... 417
Abortive Treatment of Furuncles ..... 418
Sick Headache; Bicarbonate of Sodium in the Treatment of Gonorrhœa ..... 419
Treatment of palpitation; Subnitrate of Bismuth as a Dressing ..... 420
Treatment of Dysentery; The Treatment of Rheumatism ..... 421
Hypodermic Injection of Atropine in Hæmoptysis; Aneurism Cured by Position ..... 422
Abortive Treatment of Whitlow; Benzoate of Soda in Summer Diarrhœa ..... 423
Neurotic Treatment of Catarrh; Transplantation of Tendon; Ring Pessary in Treatment of Fractured Patella ..... 424
Agaricin for Night Sweats; Indications for Thyroidectomy and for Interstitial Injections of Iodine in Parenchymatous Goitre; Cod-liver Oil in Infantile Atrophy ..... 425
The Prevention of Mammary Abscess; Reduction of Dislocation of the Humerus by Right-angle Traction; New Local Anæsthetic; Stammering ..... 426

### Scientific Items.

A Wise Wasp; How Arago Measured the Power of Steam ..... 427
Electricity a Form of Matter; High Ballooning; Artesian Well at Galveston ..... 428

### Practical Notes and Formulæ.

Ear-ache; Gonorrhœa Treatment; Iodoform in Phthisis; The Medical Treatment of Fissures in Ano by Cocaine ..... 429

Diphtheria; Parson's Local Anæsthetic; Atiseptic Mouth Wash ..... 430

A Formula for Lithuria; Duration of Infection of Eruptive Fevers; Chronic Rheumatism; Treatment of Chorea ..... 431

### Editorials and Miscellaneous.

Editorial Brevities ..... 432
The Medical Congress; Dr. Stone's Address ..... 433
Our Trip to the Medical Congress ..... 434
Books and Pamphlets Received ..... 438
Special Notes ..... 439

